# Harshlight: a "corrective make-up" program for microarray chips

**DOI:** 10.1186/1471-2105-6-294

**Published:** 2005-12-10

**Authors:** Mayte Suárez-Fariñas, Maurizio Pellegrino, Knut M Wittkowski, Marcelo O Magnasco

**Affiliations:** 1 Center for Studies in Physics and Biology, The Rockefeller University, 1230 York Ave, Box 212, New York, NY 10021, USA; 2 General Clinical Research Center, The Rockefeller University, 1230 York Ave, Box 327, New York, NY 10021, USA

## Abstract

**Background:**

Microscopists are familiar with many blemishes that fluorescence images can have due to dust and debris, glass flaws, uneven distribution of fluids or surface coatings, etc. Microarray scans do show similar artifacts, which might affect subsequent analysis. Although all but the starkest blemishes are hard to find by the unaided eye, particularly in high-density oligonucleotide arrays (HDONAs), few tools are available to help with the detection of those defects.

**Results:**

We develop a novel tool, *Harshlight*, for the automatic detection and masking of blemishes in HDONA microarray chips. *Harshlight *uses a combination of statistic and image processing methods to identify three different types of defects: localized blemishes affecting a few probes, diffuse defects affecting larger areas, and extended defects which may invalidate an entire chip.

**Conclusion:**

We demonstrate the use of *Harshlight *can materially improve analysis of HDONA chips, especially for experiments with subtle changes between samples. For the widely used MAS5 algorithm, we show that compact blemishes cause an average of 8 gene expression values per chip to change by more than 50%, two of them by more than twofold; our masking algorithm restores about two thirds of this damage. Large-scale artifacts are successfully detected and eliminated.

## Background

Analysis of hybridized microarrays starts with scanning the fluorescent image. The quality of data scanned from a microarray is affected by a plethora of potential confounders, which may act during printing/manufacturing, hybridization, washing, and reading. For high-density oligonucleotide arrays (HDONAs) such as Affymetrix GeneChip^® ^oligonucleotide (Affy) arrays, each chip contains a number of probes specifically designed to assess the overall quality of the biochemistry, whose purpose is, e.g., to indicate problems with the biotinylated B2 hybridization. Affymetrix software and packages from Bioconductor project for R [1] provide for a number of criteria and tools to assess overall chip quality, such as percent present calls, scaling factor, background intensity, raw Q, and degradation plots. However, these criteria and tools have little sensitivity to detect localized artifacts, like specks of dust on the face of the chip, which can substantially affect the sensitivity of detecting physiological (i.e., small) differences. In the absence of readily available safeguards to indicate potential physical blemishes, researchers are advised to carefully inspect the chip images visually [2,3]. Unfortunately, it is impossible to visually detect any but the starkest artifacts against the background of hundreds of thousands of randomly allocated probes with high variance in affinity.

In [4] a simple method to "harshlight" blemishes in HDONAs chips was presented. The method produces an Error Image (**E**) for each chip, which indicates the deviation of this chip's log-intensities from the other chips in the experiment. Formally, **E **is calculated as **E**^(*i*) ^= **L**^(*i*) ^- *median *_*j*_**L**^(*j*) ^where **L**^(*j*) ^is the log-intensity matrix of chip *i*. Given that the intensity of each cell is highly determined by the sequence of the probe [5], this deviation should be near zero except for the probes belonging to the probe sets related to genes that are differentially expressed. In earlier Affymetrix chips, the probe pairs corresponding to a single probeset were located in adjacent positions on the array, but now probe pairs are randomly distributed on the chip [6], so that no obvious pattern should be discernable in **E**.

In about 25% of the chips we have seen, the error image shows artifacts with strikingly obvious patterns, which often hint to the physical cause of the blemish. While this makes such blemishes visible to the human eye, manually masking the defects is impractical except for small sets of chips and introduces undesirable subjectivity. Thus, we developed an R-package with subroutines in C, to automatically spot suspicious patterns in the error image (**E**) using a battery of diagnostic tests based on both image processing and statistical approaches.

For testing and developing purposes, several sets of chips were used, including chips from Affymetrix SpikeIn (HUG133 and HUG95) experiments [6] and from three other experiments undertaken at Rockefeller University facilities, for a total of 158 chips. These include a variety of experimental sets: HGU133a chips on embryonic stem cell samples [7], two clinical studies on psoriasis [8], undertaken using blood and skin samples (Haider A., personal communication), and a study on microglia cells (Kreek M.J., personal communication).

## Implementation

**Figure 1:**
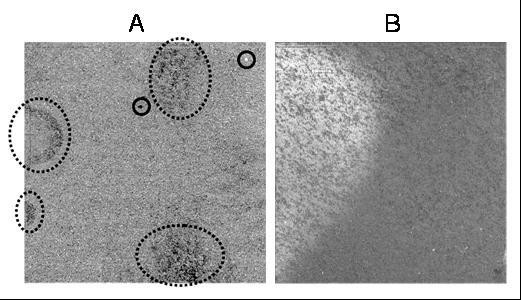
**Three types of defects**. A. Solid circles mark compact defects and dashed circles outline areas with diffuse defects. B. A chip with a large defect that invalidates its further use.

In [4] two broad categories of common defects were identified in Affymetrix GeneChips: compact and diffuse defects. **Compact defects **are characterized by a small or medium size region where all the probes are blemished, often due to mechanical and optical causes, like a piece of dirt on the face of the chip (see solid circles, Figure 1A). **Diffuse defects are **characterized by clouds with a high density of blemished probes presumably due to defects in the hybridization stage or to uneven scanner position, illumination, as those circled in dashed lines in Figure 1A. We have found evidence that these defects are probe sequence dependent, suggesting hybridization problems (see Supplementary Information: SIprobecomposition.pdf).

In this manuscript we shall also deal with **extended defects**, usually affecting a large area of the chip, as the one showed in Figure 1B. (see Ben Bolstad's homepage for more examples [9]).

We have developed pattern recognition methods specifically tailored to each type of defect. We will describe them and how they are deployed in the next sections.

### General structure

Having a batch of chips from a single experiment, the error image **E **is obtained for each chip as described above. Our algorithm detects patterns of outliers in these error images, so it is important to notice that henceforth, unless otherwise noted, we shall refer exclusively to the error images. In a typical experiment, only a small number of genes are expected to be differentially expressed. Thus, most pixels in **E **should be close to zero. Since probes belonging to a probe set are randomly distributed over the chip, variance in gene expression should not lead to spatially correlated patterns. Therefore any discernable pattern of outliers in **E **signals a defect. *Harshlight *automatically identifies those patterns and returns the batch of masked chips. The user may choose whether the intensity values of defective probes are to be substituted by missing values or by the median of the intensity values of the other chips (default).

**Figure 2:**
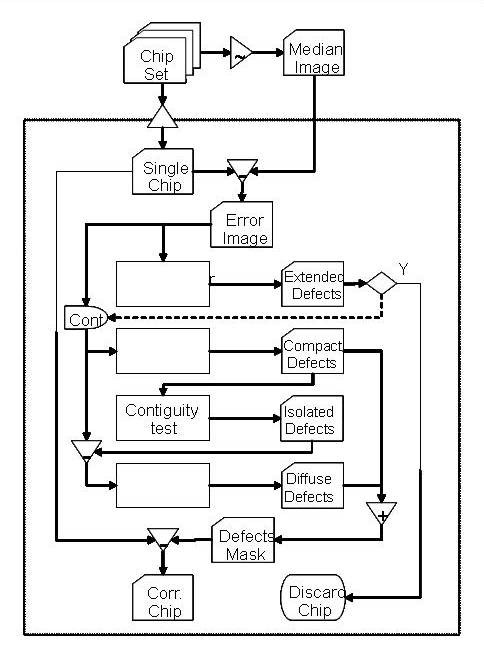
***Harshlight *flow diagram**. For each chip, an error image is obtained by subtracting the median across all chips and analyzed for the presence of extended defects. If any is found, the chip is discarded; otherwise it is searched for compact defects. Isolated compact defects are then subtracted from the error image prior to searching for diffuse defects. The expression values within compact and diffuse defects are then purged from the chip.

The program's structure is outlined in Figure 2. Initially, **E **is scanned for the presence of an extended defect and if one is found, the chip is discarded; otherwise, the routine continues by searching for compact and diffuse defects, masking them and making sure they do not belong to a diffuse defect thought a contiguity test. Once the compact defects are recognized, they are masked and the programs proceeds looking for areas of diffuse defects.

### Extended defects

Defects covering a large area (extended defects), can cause substantial variation in the overall intensity from one region of the chip to another, thereby compromising the assumption that most cells have only a small deviation from the median. To quantify such variation, we decomposed the error image **E **as:

**E **= **B**_**E **_+ **η**_**E**_

where **B**_**E **_and **η**_**E **_represent, respectively, the background and features of the error image **E. **Please note that **B**_**E **_is a background in an image analysis sense, and it should not be confused with the optical background of the original chip image that is addressed in background correction procedures; **B**_**E **_is not related to the "dark" area of the original image and in fact can have either sign. sSimilarly, the "features" of the error image are its local spatial variations, and also can have either sign. Ideally, in an unblemished chip, the features **η**_**E **_originate in differentially expressed genes, which are expected to be spatially randomly distributed with mean zero and variance σηE2
 MathType@MTEF@5@5@+=feaafiart1ev1aaatCvAUfKttLearuWrP9MDH5MBPbIqV92AaeXatLxBI9gBaebbnrfifHhDYfgasaacH8akY=wiFfYdH8Gipec8Eeeu0xXdbba9frFj0=OqFfea0dXdd9vqai=hGuQ8kuc9pgc9s8qqaq=dirpe0xb9q8qiLsFr0=vr0=vr0dc8meaabaqaciaacaGaaeqabaqabeGadaaakeaacqaHdpWCdaqhaaWcbaGaeq4TdG2aaSbaaWqaaiabhweafbqabaaaleaacqaIYaGmaaaaaa@3289@. Assuming background and features to be uncorrelated, this allows the variance of **E **to be decomposed as:

σE2=σB2+σηE2
 MathType@MTEF@5@5@+=feaafiart1ev1aaatCvAUfKttLearuWrP9MDH5MBPbIqV92AaeXatLxBI9gBaebbnrfifHhDYfgasaacH8akY=wiFfYdH8Gipec8Eeeu0xXdbba9frFj0=OqFfea0dXdd9vqai=hGuQ8kuc9pgc9s8qqaq=dirpe0xb9q8qiLsFr0=vr0=vr0dc8meaabaqaciaacaGaaeqabaqabeGadaaakeaacqaHdpWCdaqhaaWcbaGaeCyraueabaGaeGOmaidaaOGaeyypa0Jaeq4Wdm3aa0baaSqaaiabhkeacbqaaiabikdaYaaakiabgUcaRiabeo8aZnaaDaaaleaacqaH3oaAdaWgaaadbaGaemyraueabeaaaSqaaiabikdaYaaaaaa@3C6D@

To estimate σBE2
 MathType@MTEF@5@5@+=feaafiart1ev1aaatCvAUfKttLearuWrP9MDH5MBPbIqV92AaeXatLxBI9gBaebbnrfifHhDYfgasaacH8akY=wiFfYdH8Gipec8Eeeu0xXdbba9frFj0=OqFfea0dXdd9vqai=hGuQ8kuc9pgc9s8qqaq=dirpe0xb9q8qiLsFr0=vr0=vr0dc8meaabaqaciaacaGaaeqabaqabeGadaaakeaacqaHdpWCdaqhaaWcbaGaeCOqai0aaSbaaWqaaiabhweafbqabaaaleaacqaIYaGmaaaaaa@31EE@, the image is smoothed with a median filter[10], a technique commonly used in image processing to eliminate single-pixel noise. The median filtered image E˜
 MathType@MTEF@5@5@+=feaafiart1ev1aaatCvAUfKttLearuWrP9MDH5MBPbIqV92AaeXatLxBI9gBaebbnrfifHhDYfgasaacH8akY=wiFfYdH8Gipec8Eeeu0xXdbba9frFj0=OqFfea0dXdd9vqai=hGuQ8kuc9pgc9s8qqaq=dirpe0xb9q8qiLsFr0=vr0=vr0dc8meaabaqaciaacaGaaeqabaqabeGadaaakeaacuWHfbqrgaacaaaa@2DD2@, created trough a sliding median kernel, is an estimator of the background **B**_**E **_as is defined by

E˜i
 MathType@MTEF@5@5@+=feaafiart1ev1aaatCvAUfKttLearuWrP9MDH5MBPbIqV92AaeXatLxBI9gBaebbnrfifHhDYfgasaacH8akY=wiFfYdH8Gipec8Eeeu0xXdbba9frFj0=OqFfea0dXdd9vqai=hGuQ8kuc9pgc9s8qqaq=dirpe0xb9q8qiLsFr0=vr0=vr0dc8meaabaqaciaacaGaaeqabaqabeGadaaakeaacuWHfbqrgaacamaaBaaaleaacqWGPbqAaeqaaaaa@2F59@ = *median*(**E**_*j*_, *j *∊ Θ_*i*_)

where Θ_i _are the pixels in the window centered in *i*. In our case, the mask used is a circular window with a user defined radius (default = 10 pixels). At the edges of the image, the part of the mask that lies outside of the chip borders is filled with the image mirrored at the border.

Since the background is locally constant, we have approximately:

σE˜2=σBE2+ση2nδ→δ→∞σBE2
 MathType@MTEF@5@5@+=feaafiart1ev1aaatCvAUfKttLearuWrP9MDH5MBPbIqV92AaeXatLxBI9gBaebbnrfifHhDYfgasaacH8akY=wiFfYdH8Gipec8Eeeu0xXdbba9frFj0=OqFfea0dXdd9vqai=hGuQ8kuc9pgc9s8qqaq=dirpe0xb9q8qiLsFr0=vr0=vr0dc8meaabaqaciaacaGaaeqabaqabeGadaaakeaacWaLas4Wdm3aiqjGDaaaleacucOafCyrauKbaGaaaeacucOamqjGikdaYaaakiabg2da9iabeo8aZnaaDaaaleaacqWHcbGqdaWgaaadbaGaeCyraueabeaaaSqaaiabikdaYaaakiabgUcaRmaalaaabaGaeq4Wdm3aa0baaSqaaiabeE7aObqaaiabikdaYaaaaOqaaiabd6gaUnaaBaaaleaacqaH0oazaeqaaaaakmaaoGcaleqabaGaeqiTdqMaeyOKH4QaeyOhIukakiaawkziaiabeo8aZnaaDaaaleaacqWHcbGqdaWgaaadbaGaeCyraueabeaaaSqaaiabikdaYaaaaaa@5147@

where *n*_δ _is the number of pixels in the window, which is equal to 10^2^π ≈ 314 in the case of a circular window with radius δ = 10. Thus, σE˜2
 MathType@MTEF@5@5@+=feaafiart1ev1aaatCvAUfKttLearuWrP9MDH5MBPbIqV92AaeXatLxBI9gBaebbnrfifHhDYfgasaacH8akY=wiFfYdH8Gipec8Eeeu0xXdbba9frFj0=OqFfea0dXdd9vqai=hGuQ8kuc9pgc9s8qqaq=dirpe0xb9q8qiLsFr0=vr0=vr0dc8meaabaqaciaacaGaaeqabaqabeGadaaakeaacqaHdpWCdaqhaaWcbaGafCyrauKbaGaaaeaacqaIYaGmaaGccaaMcSoaaa@3245@ is a good approximation of the background variance.

In an unblemished chip, the variance of the deviations from the median chip, σE2
 MathType@MTEF@5@5@+=feaafiart1ev1aaatCvAUfKttLearuWrP9MDH5MBPbIqV92AaeXatLxBI9gBaebbnrfifHhDYfgasaacH8akY=wiFfYdH8Gipec8Eeeu0xXdbba9frFj0=OqFfea0dXdd9vqai=hGuQ8kuc9pgc9s8qqaq=dirpe0xb9q8qiLsFr0=vr0=vr0dc8meaabaqaciaacaGaaeqabaqabeGadaaakeaacqaHdpWCdaqhaaWcbaGaeCyraueabaGaeGOmaidaaaaa@30A5@, is mainly due to the variance of the features η, and the background **B**_**E **_should suffer small variations across the chip, i.e. ση2
 MathType@MTEF@5@5@+=feaafiart1ev1aaatCvAUfKttLearuWrP9MDH5MBPbIqV92AaeXatLxBI9gBaebbnrfifHhDYfgasaacH8akY=wiFfYdH8Gipec8Eeeu0xXdbba9frFj0=OqFfea0dXdd9vqai=hGuQ8kuc9pgc9s8qqaq=dirpe0xb9q8qiLsFr0=vr0=vr0dc8meaabaqaciGacaGaaeqabaqabeGadaaakeaaiiaacqWFdpWCdaqhaaWcbaGae83TdGgabaGae8Nmaidaaaaa@3133@ >> σBE2
 MathType@MTEF@5@5@+=feaafiart1ev1aaatCvAUfKttLearuWrP9MDH5MBPbIqV92AaeXatLxBI9gBaebbnrfifHhDYfgasaacH8akY=wiFfYdH8Gipec8Eeeu0xXdbba9frFj0=OqFfea0dXdd9vqai=hGuQ8kuc9pgc9s8qqaq=dirpe0xb9q8qiLsFr0=vr0=vr0dc8meaabaqaciaacaGaaeqabaqabeGadaaakeaacqaHdpWCdaqhaaWcbaGaeCOqai0aaSbaaWqaaiabhweafbqabaaaleaacqaIYaGmaaaaaa@31EE@. However, in an image such that in Figure 1B there is a large variation of the background from one region to another. Thus, the proportion of variations in **E **explained by the background, namely σBE2
 MathType@MTEF@5@5@+=feaafiart1ev1aaatCvAUfKttLearuWrP9MDH5MBPbIqV92AaeXatLxBI9gBaebbnrfifHhDYfgasaacH8akY=wiFfYdH8Gipec8Eeeu0xXdbba9frFj0=OqFfea0dXdd9vqai=hGuQ8kuc9pgc9s8qqaq=dirpe0xb9q8qiLsFr0=vr0=vr0dc8meaabaqaciaacaGaaeqabaqabeGadaaakeaacqaHdpWCdaqhaaWcbaGaeCOqai0aaSbaaWqaaiabhweafbqabaaaleaacqaIYaGmaaaaaa@31EE@/σE2
 MathType@MTEF@5@5@+=feaafiart1ev1aaatCvAUfKttLearuWrP9MDH5MBPbIqV92AaeXatLxBI9gBaebbnrfifHhDYfgasaacH8akY=wiFfYdH8Gipec8Eeeu0xXdbba9frFj0=OqFfea0dXdd9vqai=hGuQ8kuc9pgc9s8qqaq=dirpe0xb9q8qiLsFr0=vr0=vr0dc8meaabaqaciaacaGaaeqabaqabeGadaaakeaacqaHdpWCdaqhaaWcbaGaeCyraueabaGaeGOmaidaaaaa@30A5@, quantifies the extent of such defect. If this quantity is bigger than a certain threshold, the chip should be discarded.

This kind of extended defect was rare; we only found three seriously flawed chips among the 158 chips we analyzed. The percentage of the estimated variance explained by the background σE˜2
 MathType@MTEF@5@5@+=feaafiart1ev1aaatCvAUfKttLearuWrP9MDH5MBPbIqV92AaeXatLxBI9gBaebbnrfifHhDYfgasaacH8akY=wiFfYdH8Gipec8Eeeu0xXdbba9frFj0=OqFfea0dXdd9vqai=hGuQ8kuc9pgc9s8qqaq=dirpe0xb9q8qiLsFr0=vr0=vr0dc8meaabaqaciaacaGaaeqabaqabeGadaaakeaacqaHdpWCdaqhaaWcbaGafCyrauKbaGaaaeaacqaIYaGmaaGccaaMcSoaaa@3245@/σE2
 MathType@MTEF@5@5@+=feaafiart1ev1aaatCvAUfKttLearuWrP9MDH5MBPbIqV92AaeXatLxBI9gBaebbnrfifHhDYfgasaacH8akY=wiFfYdH8Gipec8Eeeu0xXdbba9frFj0=OqFfea0dXdd9vqai=hGuQ8kuc9pgc9s8qqaq=dirpe0xb9q8qiLsFr0=vr0=vr0dc8meaabaqaciaacaGaaeqabaqabeGadaaakeaacqaHdpWCdaqhaaWcbaGaeCyraueabaGaeGOmaidaaaaa@30A5@ varied across chip collections; chips handled by our local facility had a median of 3% and always had <9% variations. The SpikeIn experiments had substantially larger ratios, and in the case of SpikeIn95, three outlier chips at 33%, 36% and 60% (the chip in Figure 1B). No chip in our collection has ratios between 17% and 33%, so any number in that range seems a reasonable threshold given our limited statistics. Since typically σ_η_~0.4 in log2 units, chips with large ratios can be materially distorted; the background of the chip in Figure 1B has σ_B _= 0.5, so the intensities in the bright region are more than double the intensities in the dark area.

We do not have enough data to ascertain what causes extended defects; since the chips are scanned by a laser-scanning system, extended defects are not caused by changes in illumination level or other simple physical causes. We therefore do not currently know what an appropriate remedy would be, so if an extended defect is detected analysis is stopped for this chip, and suggest to the user the chip should be discarded.

### Compact defects

If the chip passed the previous test, analysis continues. First the chip is searched for compact defects, defined as small connected clusters of outliers in the error image **E**. As probe pairs are randomly distributed, differential gene expression leads to spatially uncorrelated variations. In good chips, the outlier pixels of **E **should not be connected, so connected outlier pixels indicate compact defects.

**Figure 3:**
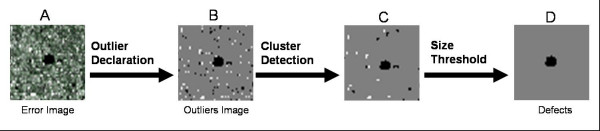
**Algorithm to detect compact defects**. Outliers (B) are declared based on the distribution function of the **E **(A). Then, clusters of outliers are identified with a FloodFill algorithm (C), and a size threshold is applied to eliminate clusters made of single or only a few pixels (D).

Figure 3 illustrates the algorithm to detect such defects. First, we declare outliers all the pixels in **E **with intensities smaller than the α-percentile (dark outliers) or bigger than (1 - α)-percentile (default: α = 2.5% both for bright and dark defects). Outlier Images are created as binary images where 1 represents pixels declared as outliers. Though in Figure 3B they are shown as a single image, dark and bright outliers are treated separately.

For each outlier image, the FloodFill algorithm[11] is then used to detect clusters of connected outliers. For every flagged pixel in the image, the algorithm recursively looks for other flagged pixels in its neighborhood. If any are found, the pixels are assigned to the same cluster number. The process stops when no more connected pixels can be found (see Figure 3C for the resulting image). The user can choose whether two pixels are considered connected if they share only an edge (4 -neighbours connectivity) or also a corner (8-neighbours connectivity, default).

**Figure 4:**
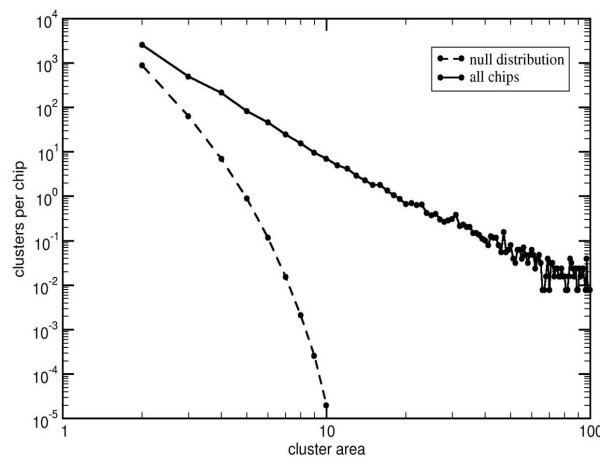
**Number of compact clusters per chip as a function of their area**. Solid curve (empirical) all chips in our collection. Dashed curve (simulation, 100000 random images) null distribution.

Even in a "good" chip, where the outliers are spatially randomly distributed, it is possible to find small clusters by chance. So, to guard against spurious results, the cluster size distribution under the null hypothesis of (spatially) randomly distributed outliers is obtained through simulation. Since this distribution depends on both chip size and density of outliers, simulations need to be carried out for each combination of those parameters. To reduce the computational burden at the procedure's runtime, we carried out simulations for common chips' designs (534 × 534, 640 × 640, and 712 × 712) and selected proportion of outliers (0.01, 0.02, 0.05, 0.10, 0.20, 0.25, 0.30, and 0.40). An example of this distribution is the lower curve in Figure 4. By default, if the user's chip dimensions or specified proportion of outliers are not among those tabulated, distribution values are interpolated from the table. However the user may override interpolation and run-time simulations will be executed.

After each cluster is defined, the significance of its size *s *can be easily computed as 1-F(*s*), where F is the cumulative distribution of cluster size under the null hypothesis in a chip of the same dimension and proportion of outliers. If the size significance is bigger than a user-defined threshold (default α = 0.01), the cluster is discarded and not considered as a blemish (Figure 3D). In addition, its size is also compared to the minimum cluster size accepted (user defined, default = 15 pixels): again, if the cluster is not large enough it is not considered. The collection of chips we have examined displays a large number of compact defects, many of them quite large.

A histogram of the size distribution of compact defects, contrasted with the null hypothesis distribution derived from simulations, is shown in Figure 4. Within the range of a few units of area through several hundred probes, the distribution of compact defects can be approximated by a power law (similar to the Zipf law in linguistics) *N*(*A*)~A^-3.1^, while the null distribution falls off exponentially as *N*(*A*)~e^-2.12A^. Therefore for even moderately small areas the significance of such clusters is extremely high.

Areas covered by compact defects are excluded from the chip before continuing with the next step.

### Diffuse defects

Diffuse defects are defined as areas with densely distributed, although not necessarily connected outliers.

**Figure 5:**
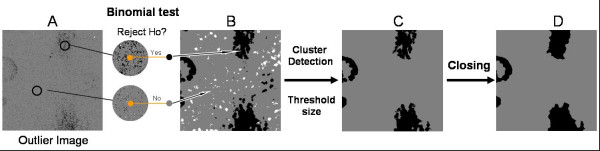
**Algorithm to detect areas with diffuse defects**. The Outlier Image is obtained as for the analysis of the compact defects. A circular kernel is then applied to each pixel in the image to detect areas in which the observed number of outliers exceeds the expected number, based on a binomial test. The defected areas thus determined undergo a round of cluster detection and size threshold, in order to eliminate small areas. The final step involves a dilation and erosion of the defects, in order to better outline the areas.

In normal chips, outliers should be spatially uniformly distributed over the image, so proportion of outliers should be similar for different regions. In case of diffuse defects, we are looking for areas in which there are a large number of outliers, when compared to other regions of the image. Figure 5 shows the algorithm used to detect areas with diffuse defects, which begins with the definition of the Outliers image.

The first step involves, as in the case of compact defects, the declaration of outliers. To avoid penalizing chips with small error variance, we declare outliers those pixels whose intensity values are higher (bright outliers) or lower (dark outliers) by a certain percentage than the expected intensity. In terms of the **E**, this criterion (also used in[12]) implies that if pixels with x% of decrease in intensities are considered dark outliers, the dark outlier image can be defined as:

Oi={1if Ei≤−log⁡2(1+x)0otherwise
 MathType@MTEF@5@5@+=feaafiart1ev1aaatCvAUfKttLearuWrP9MDH5MBPbIqV92AaeXatLxBI9gBaebbnrfifHhDYfgasaacH8akY=wiFfYdH8Gipec8Eeeu0xXdbba9frFj0=OqFfea0dXdd9vqai=hGuQ8kuc9pgc9s8qqaq=dirpe0xb9q8qiLsFr0=vr0=vr0dc8meaabaqaciGacaGaaeqabaqabeGadaaakeaacqWGpbWtdaWgaaWcbaGaemyAaKgabeaakiabg2da9maaceaabaqbaeaabiGaaaqaaiabigdaXaqaaiabdMgaPjabdAgaMjaaykW7ieqacqWFfbqrdaWgaaWcbaGaemyAaKgabeaakiabgsMiJkabgkHiTiGbcYgaSjabc+gaVjabcEgaNnaaBaaaleaacqaIYaGmaeqaaOGaeiikaGIaeGymaeJaey4kaSIaemiEaGNaeiykaKcabaGaeGimaadabaGaem4Ba8MaemiDaqNaemiAaGMaemyzauMaemOCaiNaem4DaCNaemyAaKMaem4CamNaemyzaugaaaGaay5Eaaaaaa@53C2@

Both Outlier Images (one for dark outliers, one for the bright ones, overlaid in Figure 5A), are scanned with a circular sliding window of user-defined radius (default radius δ = 10). The borders are duplicated as described for the extended defects. For every pixel *i *in the Outlier image, the proportion of outliers in the surrounding circular window Θ_i _is computed as:

pi=1nδ∑i∈ΘiOi
 MathType@MTEF@5@5@+=feaafiart1ev1aaatCvAUfKttLearuWrP9MDH5MBPbIqV92AaeXatLxBI9gBaebbnrfifHhDYfgasaacH8akY=wiFfYdH8Gipec8Eeeu0xXdbba9frFj0=OqFfea0dXdd9vqai=hGuQ8kuc9pgc9s8qqaq=dirpe0xb9q8qiLsFr0=vr0=vr0dc8meaabaqaciaacaGaaeqabaqabeGadaaakeaacaaMcSUaemiCaa3aaSbaaSqaaiabdMgaPbqabaGccqGH9aqpdaWcaaqaaiabigdaXaqaaiabd6gaUnaaBaaaleaacqaH0oazaeqaaaaakmaaqafabaGaem4ta80aaSbaaSqaaiabdMgaPbqabaaabaGaemyAaKMaeyicI4SaeuiMde1aaSbaaWqaaiabdMgaPbqabaaaleqaniabggHiLdGccaaMcSoaaa@42B3@

A binomial test is then used to decide whether *p*_*i *_is larger than the overall proportion *p*_*o *_of outliers in the image, i.e. to test *p*_*i*_>*p*_*o*_*vs. p*_*i *_= *p*_*o*_.. A new image (Figure 5B) is then created as

Di={1if pi>b1−α(po,nδ)0otherwise
 MathType@MTEF@5@5@+=feaafiart1ev1aaatCvAUfKttLearuWrP9MDH5MBPbIqV92AaeXatLxBI9gBaebbnrfifHhDYfgasaacH8akY=wiFfYdH8Gipec8Eeeu0xXdbba9frFj0=OqFfea0dXdd9vqai=hGuQ8kuc9pgc9s8qqaq=dirpe0xb9q8qiLsFr0=vr0=vr0dc8meaabaqaciGacaGaaeqabaqabeGadaaakeaacqWHebardaWgaaWcbaGaemyAaKgabeaakiabg2da9maaceaabaqbaeaabiGaaaqaaiabigdaXaqaaiabdMgaPjabdAgaMjaaykW7cqWGWbaCdaWgaaWcbaGaemyAaKgabeaakiabg6da+iabdkgaInaaBaaaleaacqaIXaqmcqGHsislcqaHXoqyaeqaaOGaeiikaGIaemiCaa3aaSbaaSqaaiabd+gaVbqabaGccqGGSaalcqWGUbGBdaWgaaWcbaGaeqiTdqgabeaakiabcMcaPaqaaiabicdaWaqaaiabd+gaVjabdsha0jabdIgaOjabdwgaLjabdkhaYjabdEha3jabdMgaPjabdohaZjabdwgaLbaaaiaawUhaaiaaykW6aaa@5781@

where *b*_α_(p,n) is the α-percentile of the binomial distribution. **D **gives a better representation of the regions with high proportion of outliers, since the disconnected pixels in the Outlier Image appear now more connected (see Figure 5B).

The FloodFill algorithm is then used to detect connected flagged pixels (**D**_*i *_= 1) as before, and clusters of small size are discarded. The user can set the size limit of the clusters, but the default value is three times the area of the sliding window.

Finally, to better outline the area of blemishes, the image undergoes a closing procedure. This is a technique commonly used in image processing to close up breaks in the features of an image (see for example [10]). In our case, a circular kernel is centered in each pixel of the image (radius = radius of the kernel used to detect the diffuse defects, see later). Its centre is flagged if any of the pixels of the kernel is flagged.

This procedure (dilation) causes the borders of the defects to grow, eventually filling empty spaces inside the features. However, in order to maintain the original outer borders of the features, another circular kernel is applied to the image. This time, the centre of the window is flagged only if all of the pixels inside the window are flagged. This procedure (erosion) reverses any extension beyond the compact hull of the original cluster.

We suggest that all probes in the closed area should be masked, but the user can choose to mask only the outlier probes.

### Contiguity test

**Figure 6:**
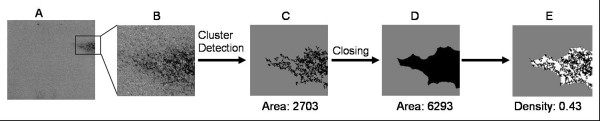
**Compact defects or part of a diffuse one?**. A. **E. **B. Zoomed area with a diffuse defect. C defects "harshlighted" as compact. D. Region delimited by the closing procedure. C. Density of the area, more than half of the probes in the region are not defective, so the region is probably considered to contain a diffuse defect.

It can happen that in a region with diffuse defects (as the one shown in Figure 6A) some blemished pixels can be clustered together, with sufficiently large size to be detected as compact defects (Figure 6C). If they were eliminated in the compact detection step, they could affect the recognition of diffuse regions. To avoid misrecognition of parts of diffuse defects as compact defects, a "contiguity test" is applied after the compact defects are detected. To perform the test, a "closing" procedure is applied to the binary image representing the compact defects (Figure 6D).

Real compact defects are isolated and highly connected clusters, so that after the closing procedure their area remains substantially the same. On the contrary, probes declared as compact defects that are part of diffuse defects are close to one another, and therefore after the closing procedure the area covered by the resulting cluster is appreciably bigger than the area covered by the compact defects alone. Comparing the extension of the areas before and after the procedure gives us an idea of how many compact defects there are in a specific region. The ratio between these two areas (Figure 6C and 6D) represents the density of compact defects in the region (Figure 6E). If this value is smaller than a threshold (default = 50%), the compact defects in the region are probably part of diffuse defects and shall not be eliminated when the compact detection procedure ends.

### Harshlight package

The package was implemented in R in compliance with the CRAN guidelines. Computationally intensive routines were implemented in C (R shared library builder 1.29 and GCC version 3.2.3) through the R interface for better efficiency.

The main *Harshlight *function accepts a Bioconductor object from the class *affyBatch*. For each batch of chips analyzed, the program returns two outputs: a file report in PostScript format and a new *affyBatch *object. The report shows, for each chip analyzed, the number and type of defects found, the percentage of the area eliminated after the analysis, an image is produced for each kind of defect, showing the areas where the blemishes are found. The output *affyBatch *object is identical to the input, except that the values within defects are declared missing. If some downstream subroutine does not allow for missing data, the user may choose to have missing data be substituted with the median of the *other *chips' intensity values for the blemished probe; this is a neutral substitution strategy, since it sets the error image values to zero on the blemished probe without affecting any other value. In general, the efficacy of an imputation method depends on what analysis is used downstream of it; because of this, only the median substitution method has been built into *Harshlight*. Other imputation methods can be still used, as functions taking an *Affybatch *object with missing values.

Other parameters to the function *Harslight *including in the implementation of the algorithm are summarized in Table 1. The choice of the default parameter values is based on our experience, but may depend on the individual lab or chip type. Still, using a uniform set of parameters for all chips in a given experiment avoids spurious effects that might be caused by manually excluding areas on the chip from analysis. Some parameters are robust, in the sense that the results are not affected by small changes. For instance, the kernel radii are robust, and so we selected the smallest value that performs well, so as to minimize running time. Other parameters, on the other hand, define the nature of what is being found; the default values we provide work well for our chip collection, and are provided as adjustable to allow for flexibility.

**Table 1:** Default values for the parameters used in the program

**Defects**	**Parameter**	**Value**
Extended	Radius of the median filter	10 pixels
	Threshold for the proportion of variance explained by the Background	30%
Compact	Quantile for the definition of outliers	5th, two tails
	Minimum size of clusters	15 pixels
	Connectivity definition	8-neighbours
	Probability value	0.01
Diffuse	Threshold for bright defects	40% more than original value
	Threshold for dark defects	35% more than original value
	p-value threshold for binomial test.	0.001
	Radius of sliding window	10 pixels
	Connectivity definition	8-neighbours
	Minimum size of clusters	3π*diff. radius

## Results and Discussion

We have built an algorithm upon a recently-developed methodology to visualize artifacts on HDONA microarrays, which automatically masks areas affected by these artifacts; we present an implementation of the algorithm in an R package, called *Harshlight*. The algorithm combines image analysis techniques with statistical approaches to recognize three types of defects frequent in Affymetrix microarray chips: extended, compact, and diffuse defects. The algorithm was tested on 158 chips, from 5 different experiments, including the two Affymetrix SpikeIn experiments. Output reports for all chips can be found online at[13].

That blemishes exist in fair abundance is clear from those output reports as well as from Figure 4. We shall now demonstrate that these blemishes affect the gene expression values and that *Harshlight *can restore this damage.

Different summarization algorithms are expected to resist blemishes differently, based on their statistical construction. We shall concentrate here on two popular algorithms, MAS5 and GCRMA. MAS5 is the "official" algorithm supplied by Affymetrix and by far the most widespread; GCRMA is an open-source method available in the Bioconductor suite, based on robust averaging techniques and sequence-dependent affinity corrections [14]. The robust averaging employed in GCRMA should confer strong immunity to outliers. We shall show below that MAS5 is strongly affected by blemishes, and that GCRMA is affected to a smaller, yet still relevant extent.

We quantified the ability of *Harshlight *to apply "corrective make-up" using two distinct strategies: first, by artificially blemishing a "clean" dataset and verifying how much the values are affected and how well they are restored, and second, by using a case where nominal concentrations are known, the Affymetrix SpikeIn experiments.

**Figure 7:**
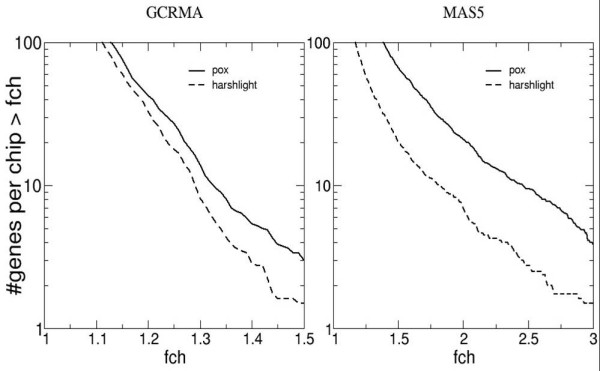
**MAS5 and GCRMA analysis**. After artificially blemishing a collection of chips we excised the blemishes with *Harshlight *using median substitution. We compare the expression values of the blemished chips vs. the original, and the expression values after restoration with *Harshlight *vs. the originals. Left, GCRMA shows a substantial improvement; right, MAS5 shows a dramatic improvement.

**Figure 8:**
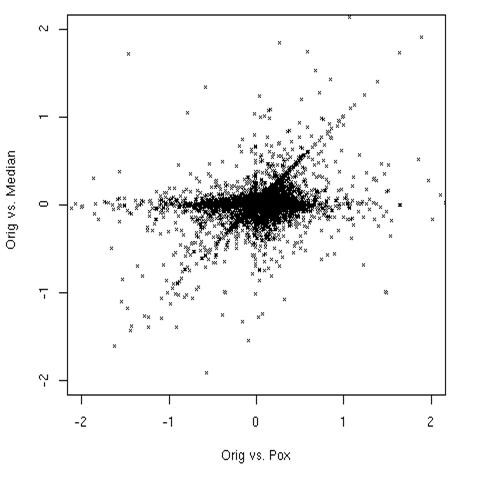
**Restoration effects**. Comparing the log fold changes in the MAS5 algorithm before and after restoration. Vertical axis, log fold change between the original expression values and the restored values (Orig vs median). Horizontal axis, log fold change between the original and blemished chips (Orig vs pox). Notice three straight lines through the plot. A diagonal line represents changed expression values that were not restored by *Harshlight*. A vertical line shows a few cases in which *Harshlight *incorrectly affected the expression values. Most points lie near the horizontal axis, showing that *Harshlight *restored values closer to the original.

For the first strategy, we wrote a simple utility we dubbed "*AffyPox*", which pockmarks a collection of chips with simulated defects with characteristics similar to those found in the test chips (Figure 4). Compact defects were simulated as randomly located circles of radius between 4 and 6 pixels, each defect having equal probability of being "bright" or "dark". The probes within the circle are linearly compressed into the lower (upper) 20% of the intensity range for dark (bright) defects. Further information on *Affypox *is available on the program's vignette. For our starting point, we took the most unblemished dataset in our collection, and we then further selected from this dataset the 8 chips with the smallest number of blemishes. Then we generated 10 artificial compact defects per chip as described above, covering less than 0.2% of the overall surface area. Since for both GCRMA and MAS5 the background and normalization process couple all the genes together, all genes' expression values are affected, most of them only by a minute amount, and a few by considerable amounts: there were 20 genes' expression values per chip affected by more than twofold in MAS5, while 3 genes per chip had more than 50% change in GCRMA. *Harshlight *detected and excised all 80 artificial defects in addition to two "false positive" defects, and reduced the number of genes affected at high fold changes, by a factor of approximately 3 in the case of MAS5, and about 2 in the case of GCRMA, as shown in Figure 7; GCRMA appears to resist blemishes better than MAS5, but at the same time the damage appears harder to undo. As with any restoration process, a large number of genes is restored, some are untouched, and a few are changed for the worse, as shown in Figure 8.

**Figure 9:**
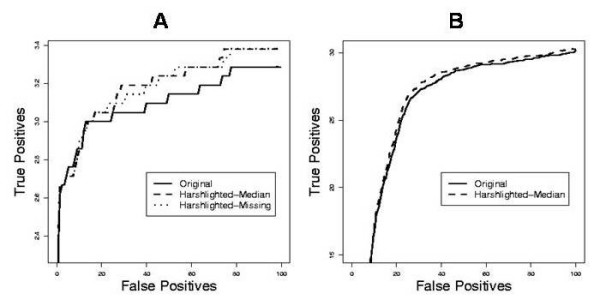
**Receiver Operator Curves generated by the *AffyComp *suite for the SpikeIn133 dataset at 2-fold changes**. A. The MAS5 algorithm shows noticeable improvement when *Harshlight *is used to excise blemishes, both for missing value substitutions as well as for median substitution: approximately 2 extra true positives per chip are discovered. B. The GCRMA algorithm shows a slight improvement of about 0.5 extra true positives per chip under median substitution.

For the second strategy, we used a well-known comparison suite, *AffyComp*, which was developed to quantify the performance of various summarization algorithms [15] on the Affymetrix SpikeIn datasets, where nominal concentrations are given for a number of genes which were "spiked in" in a Latin square experimental design. This is now an aggregate comparison, in which all defect types detected by *Harshlight *are excised, and the overall effect on the statistical performance of the summarization algorithm is quantified by means of Return Operator Curves (ROC). We performed this analysis for the most recent dataset, SpikeIn133, and again we compared the performance of MAS5 and GCRMA. We modified the MAS5 implementation in *Bioconductor *so that it accepts missing values, and compared our two substitution strategies, missing values vs. neutral replacement (i.e., substitution with the median of the other chips' values for that probe). As GCRMA is not as easily modified to allow for missing data, we could only use median substitution with GCRMA. Figure 9 shows the ROC curves summarizing the false positive/true positive behavior of the algorithm. In all cases, preprocessing the SpikeIn133 dataset with *Harshlight *results in a significant increase of performance of the algorithm, which is actually quite substantial for the case of MAS5, where >5% extra true positives are found at large false positive numbers, for both substitution methods.

In earlier chips the probesets were laid contiguously in space, so it was possible to detect localized defects by observing that the probeset had an "outlier" pattern [16]. However, the entire probeset would have to be discarded, and the gene expression information would be lost. Correlated location in space precludes use of *Harshlight *on those earlier chips (e.g., HUGeneFL). The random allocation of probe pairs in newer generations of chips permits robust methods like GCRMA to partly resist damage by blemishes; and, in conjunction with a method like *Harshlight*, to restore the expression values for many affected genes.

While we have developed this method on the basis of our error image detection of outliers, in principle the residuals of any model such as [16] or GCRMA could be used to identify individual probes on a chip as outliers. To facilitate the integration of various methods, *Harshlight *accepts error images generated by other programs; if none are provided, then the error images are computed. We have, however, not yet explored the appropriate null hypothesis for these methods.

## Conclusion

We have presented an R package that provides a way to automatically "harshlight" artifacts on the surface of HDONA microarray chips. The algorithm is based on statistical and image processing approaches in order to safely identify blemishes of different nature and correct the intensity values of the batch of chips provided by the user. The corrections made by *Harshlight *improve the reliability of the expression values when the chips are further analyzed with other programs, such as GCRMA and MAS5.

It has been shown that microarray results are affected if blemished chips enter the pipeline of the analysis; blemished probes may have values differing from the correct value by much more than the typical error, so blemishes are expected to have a particularly strong impact on experiments trying to discriminate subtle differences between samples or in a clinical diagnosis context. We present *Harshlight *in the hope it shall be a useful tool in quality assessment of microarray chips and will help improve microarray analysis.

## Availability and requirements

• **Project name: **Harshlight

• **Project home page: **

• **Operating system(s): **Platform independent, tested upon Red Hat Linux and is being under testing on Windows XP systems

• **Programming language: **R, C

• **Other requirements: **R 1.8.0 or higher

• **License: **GNU, GPL

• **Any restrictions to use by non-academics: **license needed

## Authors' contributions

MSF developed the method, designed and carried out the statistical analysis, and drafted the manuscript. MP wrote and implemented the algorithm, and helped to draft the manuscript. KMW developed the method and participated in the design of the statistical approach. MOM conceived and coordinated the study, participated in its design, and helped to draft the manuscript. All authors read and approved the final manuscript.

## Supplementary Material

Additional File 1 SIprobecomposition. nucleotide composition analysis of unblemished and blemished probes. Click here for file

Additional File 2 Harshlight R package for Linux Click here for file
