# Supplementary material for: Harshlight: a "corrective make-up" program for microarray chips
Source: BMC Bioinformatics. 2005 Dec 10;6:294. doi: 10.1186/1471-2105-6-294 (PMC1327692; doi:10.1186/1471-2105-6-294)
Supplement: Additional File 1 — SIprobecomposition. nucleotide composition analysis of unblemished and blemished probes. [file 1471-2105-6-294-S1.pdf]

Have the probes belonging to a diffuse defects different sequence-composition than the rest of the chip ?. To answer this we study the composition of the dark probes in a region with diffuse defects. Figure 1 shows that those probes have in general more amount of T and C in their sequence and less A. This effect is more clear in MM probes (Figure 1b) and still more when we compare what is the letter in the middle position of the probe (Figure 1c). We further explore this variation by position in the probe (Figure 2) and also compare how different are the affinities of those blemished probes. We can observe a strong effect in the difference in mean number of letter by position in Figure 2 (a and b). The increase in proportion of letter T and C and the decrease of A is mostly in the center probes, and not in the two ends. (Figure 2 c) shows a shifted in the affinity densities for defective probes, which is sharper in the MM case, and in the centered probes (Figure 2 d)

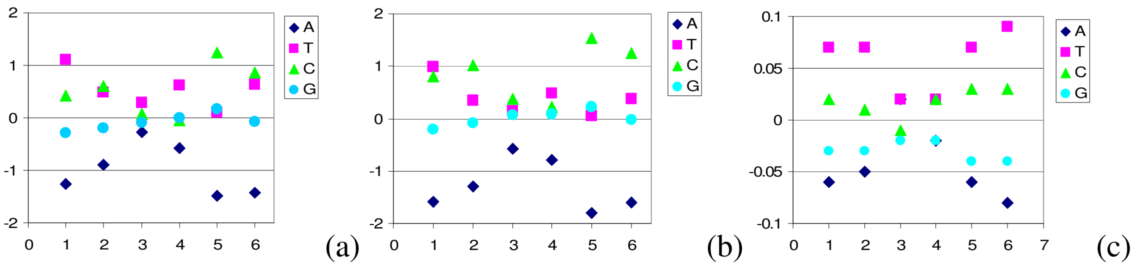

Figure 1. Differences in probe-composition between the probes belonging to diffuse defects and the whole chip. a) Differences in mean number of letters for 6 chips with difuse defects. b) as a but MM probes only. C)Diference in proportion of probes in the middle position (13).

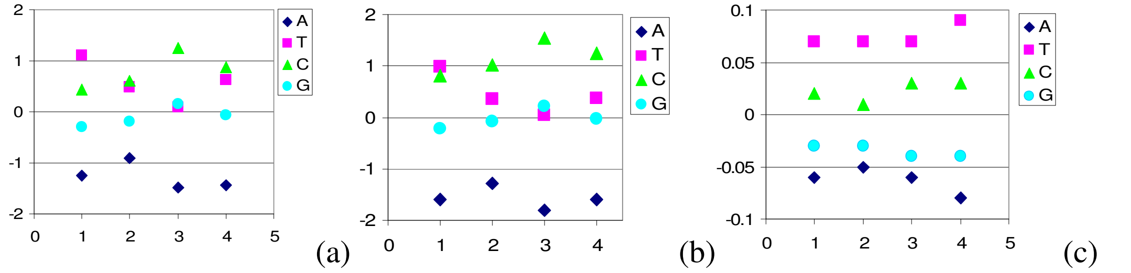

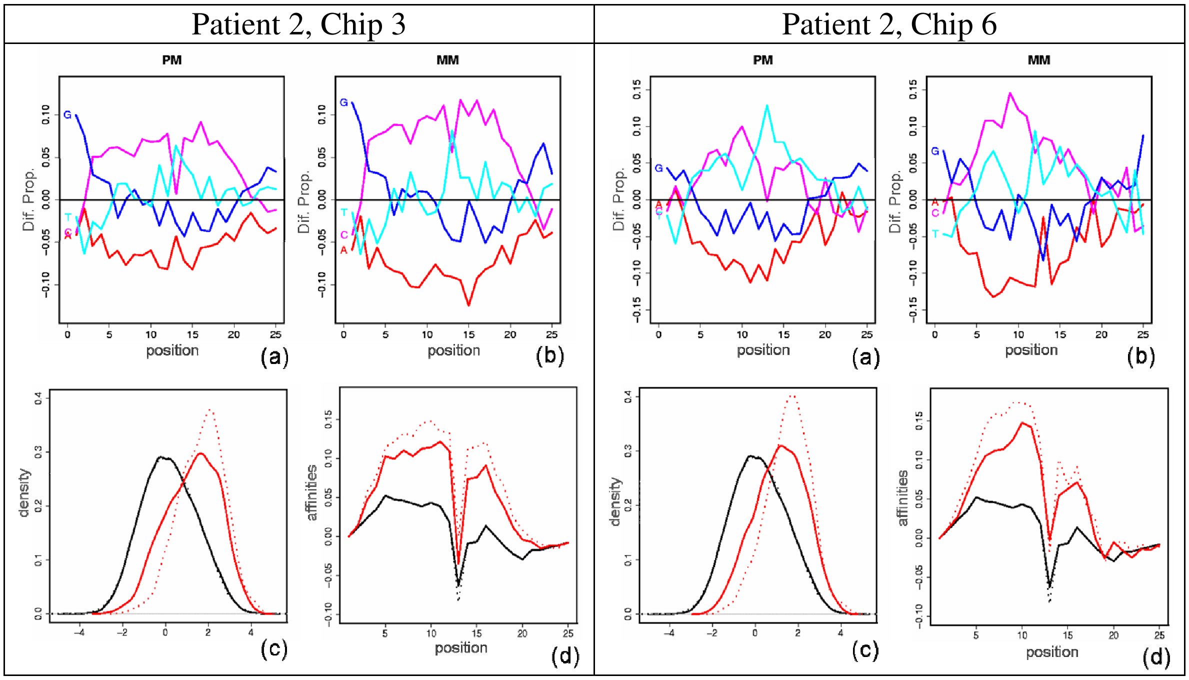

Figure 2. Comparison of the Sequence-statistics for probes with diffuse defects against all probes. 3rd and 6th chips of Patient 2. a) Difference in proportion of letter by position in the probeset a) only PM probes b) MM probes. c)Affinity densities for the general cip PM(black solid), MM (black dotted) and for probes with defects PM(red solid), MM(red dotted). d) Mean affinity by position in the probeset.
